# The effect of Polybrominated diphenyl ethers at the fetal blood-brain-barrier: evaluation using a microphysiological system

**DOI:** 10.3389/fcell.2025.1543710

**Published:** 2025-03-12

**Authors:** Sourabh Sharma, Manuel Vidal, Souvik Paul, Arum Han, Ramkumar Menon, Lauren S. Richardson

**Affiliations:** ^1^ Division of Basic Science and Translational Research, OBGYN Department, University of Texas Medical Branch at Galveston, Galveston, TX, United States; ^2^ College of Medicine, San Beda University, Manila, Philippines; ^3^ Department of Chemistry, College of Science, De La Salle University Manila, Manila, Philippines; ^4^ Department of Electrical Engineering, Texas A&M University, College Station, TX, United States

**Keywords:** fetal brain, organ-on-chip, placenta-on-chip, placenta-brain axis, glutamate

## Abstract

**Background:**

Glutamate dysregulation leading to neuronal excitotoxicity and neuroinflammation are associated with neurological disorders, specifically autism spectrum disorders (ASD) in preterm neonates. The lack of physiologically relevant *in vitro* models has limited mechanistic understanding of glutamate dysregulation and neuroinflammation during pregnancy. This study evaluated the effect of environmental pollutant and flame retardant, Polybrominated Diphenyl Ethers (PBDE) 99 and 47, on cell viability, glutamate dysregulation, and neuroinflammation using a microphysiologic system (MPS) of human fetal blood-brain-barrier organ on a chip (FB-OOC).

**Methods:**

The FB-OOC is composed of 3-cell culture chambers, connected by microchannels, containing 1) human brain microvessel endothelial cells (HBMEC), 2) human vascular pericytes (HBVP), and 3) a triculture of neurons, astrocytes, and microglia in a 5:2:1 ratio, respectively. To assess the effect of toxicants on glutamate dysregulation and neuroinflammation, control (standard media) endothelial cells were exposed to PBDE 99 and 47 (150 ng/mL). To mimic the passage of PBDE through the placenta, endothelial cells in FB-OOC were exposed to conditioned PDBE media (1:1) derived from a placenta-OOC. In parallel, triculture cells were directly treated in a 96-well plate. Dextran propagation over 72 h confirmed FB barrier function. The activation status of microglia was determined using immunocytochemistry for CD11 b and Iba1, respectively. Cell morphology (microscopy), cell cytotoxicity (Lactate Dehydrogenase and glutamate assays), and cytokines (multiplex assay) were measured.

**Results:**

Physiologic FB-OOCs were characterized by 1) viable cell cultures expressing standard cell morphologies and cell-specific markers, 2) barrier formation confirmed by decreased dextran propagation over 72 h, and 3) baseline glutamate and pro-inflammatory cytokine production. On-chip PBDE and placenta-derived metabolites of PBDE treatment in the endothelial chamber induced cell cytotoxicity and significant upregulation of glutamate in the triculture but did not induce neuroinflammation nor microglia activation compared to the controls. Conversely, 2D triculture experiments showed direct PBDE treatment-induced significant neuroinflammation (TNF-α, GM-CSF, IL-8) compared to PBDE placenta-derived metabolites or controls.

**Conclusion:**

This study established an FB model that recreated intercellular interactions. We report PBDE-induced glutamate dysregulation, often associated with the development of ASD, independent of neuroinflammation.

## Introduction

Fetal brain injury, resulting in long-term morbidity and mortality, was traditionally thought to arise from intrapartum hypoxia/asphyxia. Recent epidemiological studies have revealed that asphyxia accounts for only 20% of cases of cerebral palsy (CP). In children with CP, a potentially asphyxial birth event, inflammation, or both were experienced by 12.6%, whereas growth restriction, a birth defect, or both were experienced by 48.6% (McIntyre et al., 2013). Intraamniotic infection and inflammation (e.g., chorioamnionitis) associated with preterm birth increases the risk of neurodevelopmental disorders, including white matter injury, grey matter injury, CP, and autism spectrum disorders (ASD). Non-infectious etiologies that result in maternal systemic chronic inflammation can contribute to feto-maternal immune activation, leading to fetal neuroinflammation. Fetal neuroinflammation is also associated with attention deficit hyperactivity disorder, Tourette syndrome/chronic tic disorder (Han et al., 2021).

Although neuroinflammation is one of the underlying pathological mechanisms in toxicant exposures, the development of fetal clinical interventions is hampered on multiple levels. Understanding the cellular mechanism that results in neuroinflammation and where therapies can be applied to intervene and halt disease processes has been challenging. Human studies involving the fetus have ethical and physical challenges. Animal models are inherently costly, and the underlying mechanisms do not always translate to human disease processes (Richardson et al., 2020). Reductionist models like *in vitro* 2D and 3D models are vastly different from the *in vivo* environment, making it difficult to fully understand the various factors affecting the neurovascular unit and pathways and mechanisms contributing to neuroinflammation. These limitations also hinder the field’s ability to develop new therapeutics to treat neuroinflammation *in utero*.

The emergence of *advanced in vitro* models such as organ-on-chip (OOC) platforms can better recapitulate *in vivo* functions and responses and has the potential to move this field forward significantly. OOC technology brings together two distinct fields, microfluidic engineering and cell/tissue biology, through which diverse human organ structures and functionalities can be built into a laboratory model that better mimics functions and responses of *in vivo* tissues and organs. OOCs can overcome several of the limitations of conventional *in vitro* 2D cultures by providing a structural layout to accommodate multiple cell types in a singlex platform while allowing intercellular interaction and potential transport of secreted factors, extracellular vesicles, and even cellular migrations and communication through the chamber-to-chamber junctions. OOCs have been developed to take advantage of these characteristics to study multiple organs with initial breakthroughs in liver and lung pathologies (Ingber, 2022). Recently, multiple blood-brain-barrier (BBB)-OOCs have been created, and some have been commercialized (Nitsche et al., 2022). The preponderance of current research focuses on the adult BBB, and to date, we are not aware of any studies focusing on fetal blood-brain-barrier (FB). The primary difference between FB and adult BBB is the ongoing development throughout gestation. Initial vascularization is followed by tight junction protein and transporter expression, with further maturation with the contact between pericytes and astrocytes. The development throughout gestation improves the permeability to larger molecules through gestation and the FB response to injury (Zhao et al., 2015).

Given the variable permeability with the progression of gestation, FB may be vulnerable to environmental toxicants when compared to adjusted BBB. Polybrominated Diphenyl Ethers (PBDEs) are widely used flame retardant compounds that are persistent and bioaccumulative and, therefore, have become ubiquitous environmental contaminants. PBDE can cross the blood-brain barrier primarily due to their lipophilic nature, small molecular size, and structural similarity to thyroid hormones (Kodavanti et al., 2015). Their ability to persist and bioaccumulate in the body, coupled with the incomplete formation of the blood-brain barrier during early development, further facilitates their entry into the brain, potentially leading to neurotoxic effects. Animal studies suggest that prenatal PBDE exposure may result in adverse neurodevelopmental effects, including long-lasting behavioral alterations, particularly in the domains of motor activity and cognitive behavior (Costa and Giordano, 2007). There is also epidemiological data demonstrating neurodevelopment effects, lower on tests of mental and physical development at ages 12–48 and 72 months in relation to high cord blood PBDE concentrations (Herbstman et al., 2010). Unraveling the underlying mechanism is crucial to further our understanding of toxicant exposure in the prenatal period, improving our diagnostic abilities, and advancing therapeutic development.

This study evaluated the effect of PBDE 99 and 47 using a previously validated 3-chamber OOC platform reconfigured to contain fetal blood-brain-barrier (BBB) and fetal brain tri-culture. PBDE induced glutamate dysregulation but did not cause neuroinflammation (Table 1). The 2D *in vitro* studies and the direct toxicant exposure on-chip do not model physiologic fetal brain exposures *in utero*. To best model exposure risks FB, cells in FB-OOC should be exposed to metabolites of PBDE after their propagation and processing by the placenta.

**TABLE 1:** A summary table listing results in FB-OOC following treatment with all toxicants and responses noted in different assays.

	PBDE			PLA-OOC PBDE			CSE		
	Endothelial	Pericyte	Triculture	Endothelial	Pericyte	Triculture	Endothelial	Pericyte	Triculture
Glutamate			↑	↑		↑			↑
Inflammation			↑				↑		↑
Glial Activation									↑

## Methods

### Cell lines

All cell lines were obtained commercially and were maintained according to manufacturer instructions. All cell cultures were maintained in a 37°C, 5% CO_2_ incubator, with fresh media replaced every 48–72 h. Upon reaching 70%–90% confluence, cells were passed into fresh culture flasks. The fetal brain cells used in this study were SVG p12 (astroglia) obtained from ATCC (ATCC cat. no. CRL-8621, Manassas, VA) and HMC3 (microglia) (ATCC cat. no. CRL-3304, Manassas, VA). Astroglia and microglia were maintained ATCC-formulated Eagle’s Minimum Essential Medium (cat. no. 30–2003) with 10% fetal bovine serum. SH-SY5Y (neuroblastoma cells) obtained from ATCC (cat. no. CRL-2266) represented neurons. Neuroblastoma cells were maintained in Dulbecco’s Modified Eagle Medium (Corning cat. no. 10092CV, New York, United States) with 10% FBS. Human vascular pericytes (HBVP) and human brain microvascular endothelial cells (HBMEC) were obtained from Sciencell (cat. no. 1200 and 1000, respectively). HBVP were cultured in pericyte media (Sciencell cat. no. 1201) in flasks and chambers coated with Poly-L-Lysine (Sigma cat. no. P4707) at 2ug/cm^2^. HBMEC were cultured in Endothelial cell medium (Sciencell cat. no. 1001) in flasks and chambers coated with fibronectin (Sigma cat. no. 341631) at 2ug/cm^2^.

### Creation of the FB-OOC

**FIGURE 1:**
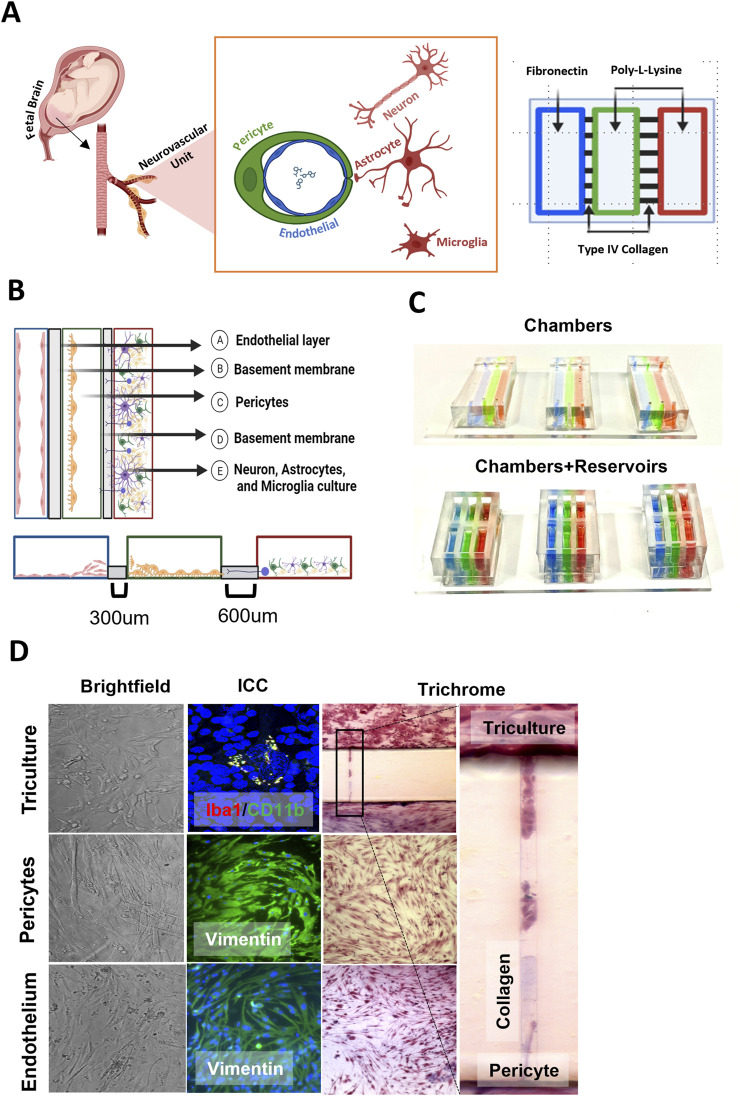
Validation of FB-OOC device. **(A)** Anatomic scheme for the design of the chip, highlighting the translation from anatomy to OOC design. **(B)** All microchannels are coated with collagen IV and chambers with cell specific basement membrane molecules to aide with cell adhesion. **(C)** Chambers are color coded for visualization. **(D)** Brightfield images of seeded cells, showing cells growing in each cell culture chamber. Immunofluorescence images after staining with markers to demonstrate makers of activated/inactive microglia, cell morphology, and cytoskeletal proteins. Visualization of cell migration between compartments through the use of Masson trichome stain as a counter stain.

The microfabrication procedure is similar to the one described previously, and the 3-chamber OOC model has been previously established (Richardson et al., 2022). The design dimensions for the chip are shown in Figure 1. The FB-OOC is composed of three poly (dimethylsiloxane) (PDMS) cell culture chambers; each cell chamber is 250 μm in height and 2,000 μm in width. The microchannels between the left chamber containing HBMEC and the middle chamber containing HBVP measured 300 um (length: 300 μm; width: 30 μm; height 5–10 μm). The microchannels connecting the middle chamber to the right chamber, containing the brain triculture of cells, measured 600 um (length: 600 μm; width: 30 μm; height 5–10 μm). To summarize, a 2-step photolithography process was conducted using a photosensitive epoxy (SU-8; MicroChem, Westborough, MA, United States) to fabricate the master mold. A soft lithography technique was utilized to make the OOC out of polydimethylsiloxane (PDMS). PDMS devices were replicated from the master mold by pouring PDMS prepolymer (10:1 mixture, Sylgard 184; DowDuPont, Midland, MI, United States) onto the mold, followed by curing at 85°C for 45–60 min. This on-chip reservoir block is created in PDMS using a similar soft lithography process as described above but using a CNC-milled acrylic layer as the master mold from which the PDMS device was replicated. This PDMS layer was treated with oxygen plasma (Harrick Plasma, Ithaca, NY, United States) for 90 s to improve the bonding of the PDMS layer onto the glass substrate and also to make the device hydrophilic for easy cell and culture medium loading. Three OOCs were bonded to a single microscope slide.

### Basement membrane loading in the FB-OOC

Before using the FB-OOC, the devices were washed with 70% ethanol for 15 min for sterilization, washed 3 times with 1X PBS, and then the microchannels were coated with Type 4 collagen (Corning Matrigel Basement Membrane Matrix, DEV-free; 1:4 in SF-media) by loading collagen into the pericyte chamber and applying suction pressure from the endothelial or brain triculture chamber through a 200 μL pipette tip attached to a vacuum system. Excess Type 4 collagen was removed from the chambers. Subsequently, the endothelial chambers were coated with Poly-L-Lysine (2ug/cm^2^) [Sigma-Aldrich, Cat. no. P4707-50 ML]. The pericyte and brain triculture chambers were coated with bovine plasma fibronectin (2 μg/cm^2^ of surface area) [ScienceCell, Cat# 8248]. The reservoirs were filled with cell-specific appropriate media. The devices were then incubated at 37°C in a 5% CO2 environment for 4 h prior to cell seeding.

### Cell seeding/culture in the FB-OOC and 2-D culture

Cells were passaged, and counted, and necessary volumes were pipetted to reach a predetermined number to reach >70% confluence per manufacturer instructions. For OOC experiments, the number of cells seeded are as follows: 120,000 endothelial cells, 120,000 pericytes, and brain triculture comprised of 37,500 neurons, 15,000 astrocytes, and 7,500 microglia for a total cell volume of 60,000 in a 5:2:1 ratio. In parallel, the brain triculture alone was seeded in a 96-well plate for direct exposure to toxicants. HBVP was introduced into the middle chamber. The slides were placed at an acute angle against a heavy wall beaker to facilitate attachment of HBVP over the microchannels facing the HBMEC chamber for 1 h and incubated at 37°C in a 5% CO2. At this time, appropriate media was placed within each reservoir, and the devices were incubated overnight at 37°C in a 5% CO2 environment. The following day, following a similar protocol, the HBMECs were introduced to the left chamber and incubated at an acute angle, facilitating the attachment of HBMECs over microchannels facing the left chamber. The devices were incubated at 37°C in 5% CO2 for 1 h. Following the incubation period, the brain triculture cells were loaded into the right chamber. Rotation of the device during seeding allows for endothelial cells and pericytes to grow over the microchannels as well as on the bottom of the chamber; forming a 3D and 2D growth. The brain triculture grew on the bottom of the chamber and grew into the microchannels. The reservoirs were filled with 75-100ul of appropriate media, and devices were incubated at 37°C in a 5% CO2 for 6 h, following which treatment was introduced.

### Cigarette smoke extract

Water soluble cigarette smoking extract (CSE) preparation was adapted as previously reported by bubbling smoke drawn from a single-lit commercial cigarette that represented high tar (unfiltered Camel; R.J. Reynolds Tobacco Co., Winston-Salem, NC) through 25 mL of HBMEC media (Menon et al., 2013). The treated HBMEC media was sterilized by using a filter of 0.2 µm (Corning, New York, United States). Next CSE was diluted to 1:50 HBMEC media for use. CSE was used as a potent oxidative stress inducer (Menon et al., 2013; Bredeson et al., 2014; Menon et al., 2014; Menon, 2022; Richardson et al., 2023) in this system to create a diseases state. A gradient was established in the reservoir block by differential volumes of cell culture media across the different chambers (120 μL–HBMEC, 100 μL–HBVP, 80 μL–Triculture). The cells were treated for 72 h at which point the supernatant and chamber content were aspirated and stored at −80 C for various assays.

### Polybrominated diphenyl ethers (PBDE) 99 and 47 exposure

Seeded cells were treated with either 150 ng/mL of PBDE-47 (Sigma) and PBDE-99 (Sigma) or conditioned media from placenta-OOC (1:1). Conditioned media was collected following PDBE 99/47 treatment within a placenta-OOC model as published previously (Vidal et al., 2024). Briefly, the authors used an OOC with seven interconnected chambers and cell types (decidua, placental vessel, cytotrophoblast, syncytiotrophoblast, placental stroma, umbilical vein endothelium) representing the placenta. PBDE 99 and 47 (150 ng/mL) were added to the placental vessel chamber, and cells were treated for 48 h using a differential volume gradient used in the current study. The media was collected from the fetal collection chamber representing placental processed PBDE and produced metabolites that crossed the entire placenta villous tree and that the fetus would be exposed to. PBDE and PBDE-condition media were dissolved in HBMEC media (1:1) for OOC testing. Treatment was conducted in a similar fashion as described above.

### Dye propagation

Barrier function testing was completed using dye propagation utilizing 3,000 kDa Dextran beads (D3305, Invitrogen, Waltham, MA) in FB-OOC with extracellular matrix alone and extracellular matrix with cells seeded. The dye was introduced to the HBMECs of the FB-OOC and diluted in chamber-specific media. A diffusion gradient was created to facilitate dye propagation (120 μL–HBMEC, 100 μL–HBVP, 80 μL–Triculture). At the end of the 72-h, fluorescence was measured by collecting images and quantifying mean intensity using ImageJ.

### Masson trichrome staining

Briefly, separate devices were stained with Masson trichrome stain to image type IV collagen inside the microchannels. To show that our matrix loading into the microchannels was evenly distributed, devices were rinsed with PBS and fixed at room temperature with 4% paraformaldehyde for 20 min. The devices were then stained with Biebrich scarlet-acid fuchsin for 10 min and then rinsed with water three times. This process stained all the cells and collagen a red color. Next, phosphomolybdic-phosphotungstic acid was applied for 15 min, which removed the red stain from the collagen. Aniline blue solution was then added for 10 min to stain the collagen a blue color. Once the device was stained, it was rinsed three times with water and imaged. This procedure was also carried out on some devices after 72 h of cell culture to monitor the collagen degradation caused by cell migration.

### Immunocytochemistry

Immunocytochemical staining markers were performed after 72 h, as previously reported. Vimentin (Abcam cat. no. ab92547) was used to visualize the morphology of HBMEC and HBVP. Lastly, a qualitative analysis of activated versus inactive microglia was completed using IBA1 (Abcam cat. no. ab221003) and CD11b (Abcam cat. no. ab197701). For each condition (control, CSE, PBDE, or PBDE PLA-OOC) N3 of staining was conducted for Iba1 and CD11b. In each N, 2-4 regions were imaged spaced out from the top–bottom of the chamber. Each image microglia were identified (globular shape, thick processes, and large nuclei) (average 1-2 cells per filed) and counted up to 15 cells. Manufacturer’s instructions were followed to determine optimum dilution to ensure uniform staining. After 72 h, cells were fixed and permeabilized with 70% ethanol and blocked with 3% bovine serum albumin in PBS prior to incubation with primary antibodies overnight at 4°C. The vimentin primary antibody was diluted to 1:200 in 3% bovine serum albumin in PBS. Iba1 and CD11b were diluted to 1:100 and 1:500 in 3% bovine serum albumin in PBS. After washing with PBS, the devices were incubated with appropriate secondary antibodies at a 1:1000 dilution in 3% bovine serum albumin for 1 h in the dark. The devices were washed with PBS, treated with NucBlue Live ReadyProbes Reagent (Thermo Fisher Scientific), and imaged accordingly. Primary and secondary concentrations were validated based on previous chip-based studies.

### Microscopy

#### Bright-field microscopy

Images were captured using a Nikon Eclipse TS100 microscope (4×, 10×, 20×) (Nikon). Three regions of interest per condition were used to determine the overall cell morphology.

#### Fluorescence microscopy

Following immunocytochemistry, a Keyence All-in-one Fluorescence BZ-X810 microscope (4×, ×10, and ×40 magnification) was used for photodocumentation.

### Cytotoxicity testing

Cytotoxicity post-seeding was determined using a commercial lactate dehydrogenase (LDH) cytotoxicity detection kit [Roche, Cat# 11644793001, Mannheim, Germany]. LDH is a relatively stable protein found in the cytoplasm, and this enzyme is released once any leakage occurs in the plasma membrane, usually because of cellular damage leading to a cell death phenotype (apoptosis, necrosis, and other forms). Cell culture media from each chamber of the FB-OOC was collected 72 h after treatment. Approximately 10 μL of cell supernatants were used to perform the cytotoxicity assay according to the manufacturer’s protocol.

### Multiplex assays for pro- and anti-inflammatory cytokines

Cytokines and chemokines (granulocyte-macrophage colony-stimulating factor (GM-CSF), interleukin (IL)-8, tumor necrosis factor-alpha (TNF-ɑ) were analyzed from the cell supernatants in the FB-OOC after treatments to investigate changes using multiplex assay kits (Milliplex, Sigma). Supernatants were manually collected from the reservoirs of all devices after 72 h of treatment. Standard curves were developed with duplicate samples of known quantities of recombinant proteins that were provided by the manufacturer. Sample concentrations were determined by relating the fluorescence values that were obtained to the standard curve by linear regression analysis. Raw data collected in pg/mL was normalized to controls from each plate prior to final analysis and representation in a heat map.

### Glutamate assay

The secreted levels of Glutamate were measured by a Glutamate Assay Kit (Fluorometric) (Abcam; ab138883). The fluorescence-based test “Glutamate Assay Kit (Fluorometric)” was used according to the protocol recommended by the manufacturer. Briefly, supernatant was collected for FB-OOC and 50 μL was added to 96-well black-walled plates along with 50 μL of Glutamic acid Reaction Mix prepared according to manufacturer’s instructions. The plate was incubated at room temperature for 90 min and fluorescent intensity of excitation λ = 550 nm, emission λ = 590 nm was measured.

### Statistical analyses

The data were expressed as the mean ± standard deviation (SD). Unpaired, Student’s t-tests were used to compare two groups. Though cell viability and glutamate results are shown as graphs containing all cell type and treatment values, cell-to-cell differences were not evaluated, statistical analysis was only conducted within each cell type between control and individual treatments. All experiments were conducted multiple times (N3-9) depending on the endpoint of interest and graphs plotted using Prism 8 software (GraphPad Software, La Jolla, CA, United States).

## Results

### Establishing a novel FB-OOC model

The FB-OOC design facilitates the movement of compounds from fetal vascular through the various cells forming the BBB to the brain triculture of neuron and glial cells (Figure 1A). The addition of cell-specific adhesion components into each chamber provides a basement membrane to promote cell adhesion (Figure 1B). The FB-OOC is composed of three chambers in series. The left chamber (marked in blue in Figure 1C) contains the HBMECs, the middle chamber (marked in green in Figure 1C) contains the HBVP cell culture, and the rightmost chamber (marked in red in Figure 1C) contains the brain triculture. These chambers were connected by a series of microfluidic channels filled with type IV collagen where cells can migrate (Figure 1D). The three culture compartments connected by microchannels can maintain their respective culture conditions.

The developed FB-OOC provided an environment that was conducive to the growth and proliferation of various cell lines utilized for 72 h. The cells demonstrated appropriate morphology in each chamber (Figure 1D). HBMEC demonstrated endothelial morphology with polygonal cell shapes and well-defined cell boundaries (Figure 1D–Endothelium), and morphology was further confirmed when staining with vimentin. Pericytes in the FB-OOC were noted to have oval cell bodies with large primary protrusions, and this morphology was confirmed by vimentin staining. Lastly, the brain triculture containing all three populations was confirmed under light microscopy. Additionally, the presence of activated (Iba1+/CD11b+) and inactivated microglia (Iba1+ or CD11b+) was documented (Figure 1D–Triculture). Their results validate that the cellular components retain their baseline characteristics within the FB-OOC.

### Diffusion dynamics across the FB-OOC

**FIGURE 2:**
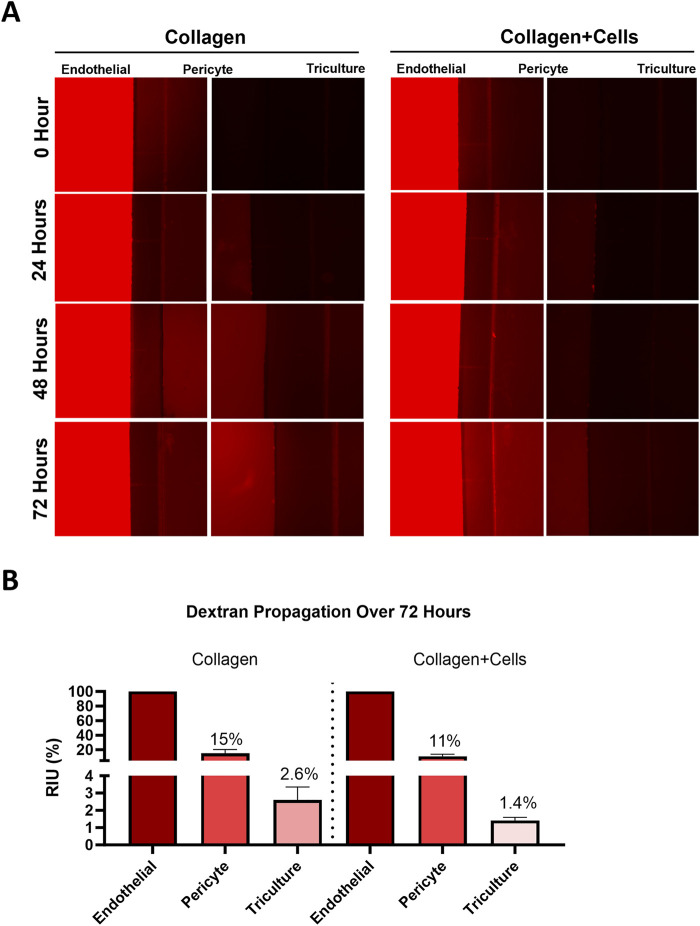
Fluid dynamics. **(A)** Dye propagation was evaluated for 72 h on-chip by adding 3,000 kDa dextran beads in the HBMEC chamber. Fluorescent images were captured over time to document bead propagation across different layers of the FB-OOC with and without collagen + cells. **(B)** Bar graph showing mean fluorescence intensity for each chamber normalized to the endothelial cell chamber.

To determine the flow of materials and their kinetics, as well as to measure cell barrier function, dextran beads (molecular weight = 3,000 kDa) were utilized to determine hydrostatic pressure across the device. A cellular barrier was formed and confirmed using light microscopy, showing the confluence level to cover the microchannels sufficiently. Functional barrier testing was conducted via 3,000 kDa Dextran bead propagation and was introduced to the endothelial chamber for 72 h. Fluorescence was measured from the captured images and normalized to control devices without Dextran beads. A barrier formation was confirmed with light microscopy and validated by Dextran bead propagation assay that showed a 1.9-fold decrease in fluorescence in the tri-culture in FB-OOCs with cells when compared to Type IV collagen (Figures 2A, B). Overall, these results demonstrate the presence of a weak cell barrier in the FB-OOC.

### Establishment of an oxidative stress-induced disease state

**FIGURE 3:**
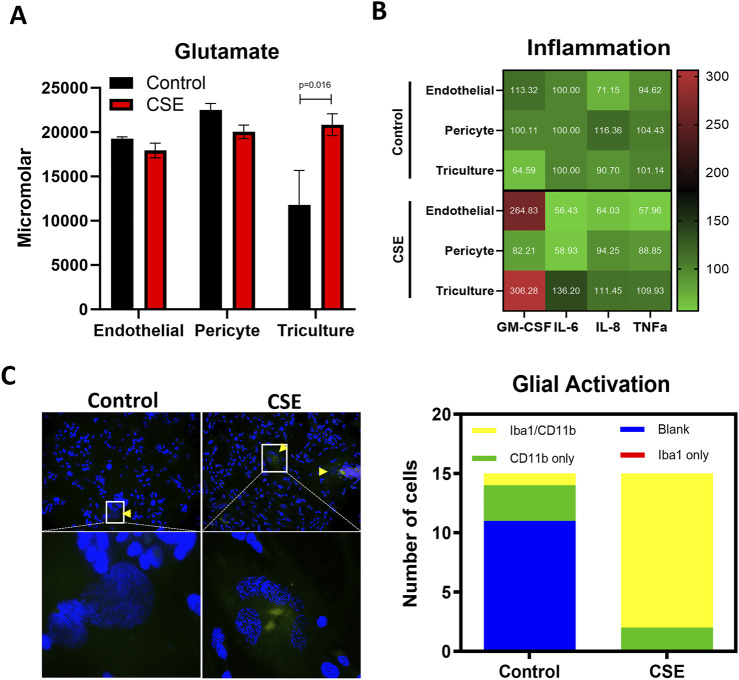
Establishment of an CSE-induced oxidative stress-induced disease state. **(A)** Glutamate assay showing the increased neuronal dysfunction in response to CSE within the triculture as compared control media. T-test was conducted between control and individual treatments to determine statistical significance. **(B)** Pro-inflammatory cytokine analysis showed in a normalized heatmap highlighting the inflammatory shifts after each treatment and within each cell type. Values are normalized to the control average and shown in pg/mL. **(C)** Qualitative analysis of glial cells stained with CD11b and/or Iba1 demonstrating a shift towards activated microglia as compared to control.

Maternal inflammation during pregnancy has been linked to OS in the fetal brain (Goeden et al., 2016; Ginsberg et al., 2017), which can impair BBB development and function. Excessive production of reactive oxygen species (ROS), nitric oxide (NO), and peroxynitrite can disrupt tight junctions and cause cytotoxic effects, leading to BBB injury. PBDE 99 and 47 is expected to induce OS. Therefore, OS was induced in the FB-OOC to stimulate a diseased state and establish pathologic criteria for comparison purposes (positive control). Cigarette smoke extract (CSE) has been utilized extensively as a proxy for inducing OS (Menon et al., 2013). To establish this, we used diluted CSE in HBMEC media (1:50), which has been shown to induce inflammation at the feto-maternal interface. Following treatment, glutamate dysregulation was noted in the brain triculture (*p = 0.016*) when compared to control (Figure 3A). No difference was noted in the endothelial and pericyte chambers. Of the markers of inflammation, pro-inflammatory cytokine GM-CSF was noted to be elevated in the brain triculture and endothelial following treatment with CSE compared to control (>2-fold increase) (Figure 3B). No differences were noted with other cytokines/chemokines, including IL-6, IL-8, and TNF-alpha. Lastly, we qualitatively examined the presence of activated versus inactivated microglia following treatment with CSE versus control media. Treatment with CSE demonstrated a higher proportion (>2-fold increase) of microglia with combined markers (Iba1/CD11 b) versus with control media, which demonstrated a higher proportion of markers of inactivated microglia (CD11 b or Iba1) or no markers (Figure 3C). These results define OS-induced neuroinflammation on-chip as increased glutamate dysfunction, an increase in pro-inflammatory cytokine production, and a shift in glial activation status.

### Evaluation of PBDEs effects at the FB

**FIGURE 4:**
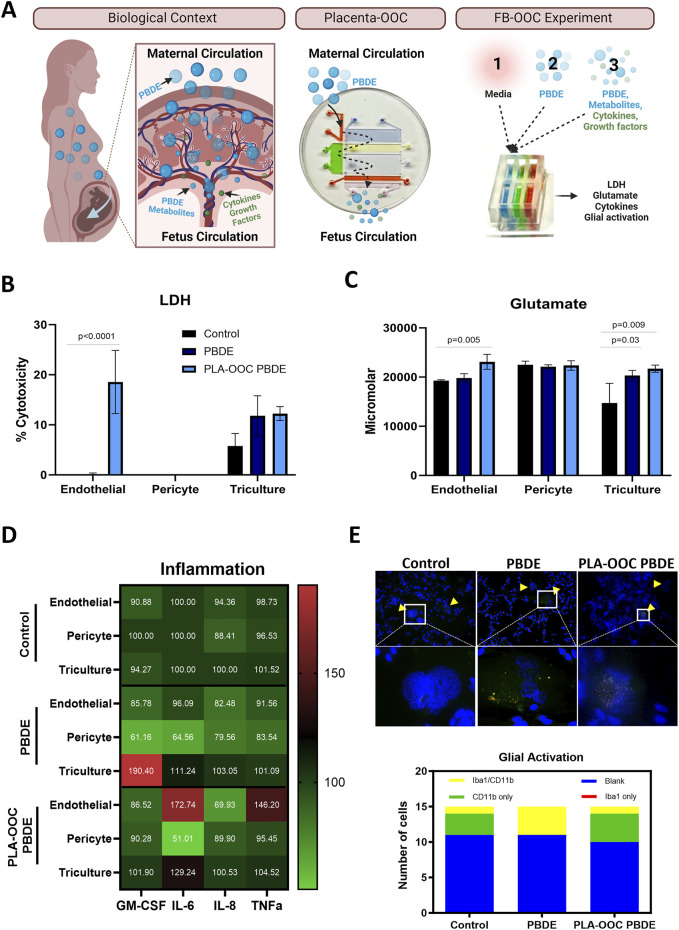
Determining the effect of PBDE at the fetal brain interface. **(A)** Experimental design demonstrating the real-world exposure of fetus to PBDE-metabolites through placental processing and generation of metabolite. PLA-OOC showing introduction of PBDE into placental vessel chamber and collected from the umbilical vein endothelium chamber containing metabolites of PBDE. Lastly schematic of the FB-OOC experiment introducing toxicants into the HBMEC chamber in the FB-OOC. **(B)** LDH assay showing percent cytotoxicity in response to PBDE and PDBE-conditioned media relative to control following 72 h of treatment only affecting endothelial cells. T-test was conducted between control and individual treatments to determine statistical significance. **(C)** Glutamate assay showing the increased neuronal dysfunction in response to PBDE and PBDE conditioned media with efflux to the endothelial chamber in response to PBDE-conditioned media. T-test was conducted between control and individual treatments to determine statistical significance. **(D)** Pro-inflammatory cytokine analysis showed in a normalized heatmap highlighting the inflammatory shifts after each treatment and within each cell type. Values are normalized to the control average and shown in pg/mL. **(E)** Qualitative examination of the microglia population demonstrates a shift towards activation, with a higher proportion cell expressing Iba1/CD11b markers versus CD11b or Iba1 alone in the control treatments.

The association between PBDE exposure and negative neurodevelopmental effects has been documented in animal and epidemiological data (Costa and Giordano, 2007; Herbstman et al., 2010). This effect has been demonstrated in human studies for up to 72 months, demonstrating lower scores on mental and physical development tests. However, there is a lack of understanding of the underlying mechanism. PDBE exposure is ubiquitous and passive. We plan to study both the biological context and direct effect of PDBE (Figure 4A). The biological context in which a patient is exposed to PBDE from within the environment and PDBE is processed by the placenta and its direct metabolites and other factors (growth, cytokines/chemokines, *etc.*) generated by placental processes are introduced to the FB in our OOC. These are represented by conditioned media acquired from PLA-OOC experiments reported elsewhere (Vidal et al., 2024). Direct exposure of PBDE was also studied by introducing PBDE within the FB-OOC and in a 2D culture directly to the brain triculture.

#### The direct effect of PBDE at the FB

**FIGURE 5:**
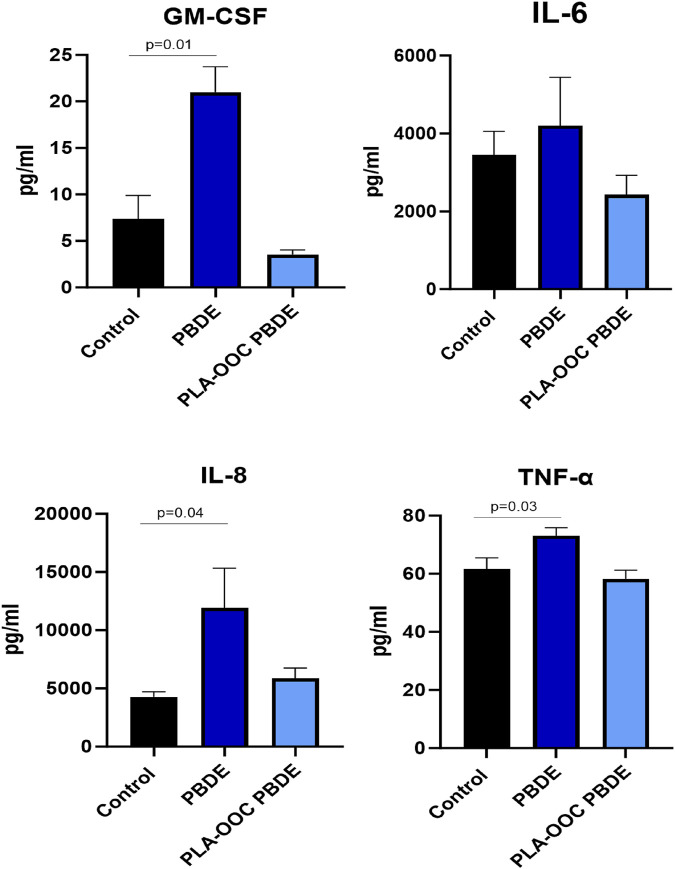
Evaluation of PBDE in 2D culture. Cytokine assay in 2D experiment with direct exposure of triculture to toxicants. Neuroinflammation, with elevated GM-CSF, IL8, and TNF-alpha, in response to PBDE but not PBDE-conditioned media as compared to control. T-test was conducted between control and individual treatments to determine statistical significance.

HMBEC were exposed to PBDE in the OOC, and treatment was continued for 72 h. Treatment with PBDE did not elicit changes in cellular toxicity in the endothelial, pericyte, or brain triculture chambers (Figure 4B). However, glutamate dysregulation, representing neuronal dysfunction, was noted with PBDE in the FB-OOC in the brain triculture (*p = 0.03*) when compared to the control conditions but not in the endothelial or pericyte chambers (Figure 4C). Proinflammatory cytokine GM-CSF was noted to be elevated in the brain triculture (>2-fold increase) (Figure 4D). Lastly, qualitative analysis examining the microglia population demonstrated no change in Iba1/CD11 b expression compared to controls (Figure 4E). In contrast, when brain triculture was directly introduced to PBDE in 2D experiments, there was a significant shift to a pro-inflammatory environment (GM-CSF (*p = 0.01*), IL-8 (*p = 0.04*) and TNF-alpha (*p = 0.03*) that was not observed on-chip (Figure 5).

#### The indirect effect of PBDE and the role of the placenta in regulating FB neuroinflammation

Next, to evaluate the effect of PDBE exposure within the biological context, PDBE-conditioned media collected from the fetal outlet chamber of the PLA-OOC, containing PDBE metabolites and PBDE from the PLA-OOC, was introduced to HBMEC, and treatment completed for 72 h (Figure 4A–FB-OOC Experiment). Conditioned media elicited cellular toxicity represented by elevated levels of LDH in endothelial cells (*p < 0.0001)* but not in the pericytes or brain triculture (Figure 4B). Glutamate efflux was noted in the brain triculture, representing neural toxicity, in response to PBDE-conditioned media. Higher levels of glutamate were also detected in the endothelial chamber when compared to control media (*p = 0.005*) (Figure 4C). In the 2D experiments, the exposure of brain triculture to the PLA-OOC PBDE-conditioned media did not demonstrate an inflammatory response (Figure 5). Similarly, within the FB-OOC, no inflammation was noted in the brain triculture. However, endothelial cells demonstrated a trend towards an inflammatory response, elevated IL-6 and TNFα, to exposure to PLA-OOC PBDE placenta metabolites, however, not reaching the 2-fold threshold (Figure 4D). The qualitative evaluation of the microglia population demonstrated populations similar to the control (Figure 4E). Exposure of brain triculture to PDBE-conditioned media from the PLA-OOC resulted in cellular and neural toxicity, as indicated by elevated LDH levels and glutamate efflux. Still, no significant inflammatory response was observed, except for endothelial and pericyte inflammation due to PDBE placenta metabolites.

## Discussion

Neuroinflammation has been thought to be a core mechanism in the development of fetal brain injury. However, the development of therapeutics has been hampered by the limitations of models. Reductionist models, such as cell culture, do not account for the cellular complexity that modulates insult responsiveness and produces results *in vivo*. Animal studies have ethical and financial challenges. Additionally, therapeutics developed in animal models do not translate to human disease processes.

We have developed a “fetal blood-brain-barrier model”, and 1) evaluated the effect of compounds that are filtered through a placenta to mimic an *in-utero* environment, 2) the FB-OOC allowed the diffusion of molecules mimicking the weaker fetal blood-brain-barrier as *in utero*, 3) embryonic derived microglia and astroglia were used to model the fetal brain, and 4) this device contained a majority of the cellular and collagen layers of the BBB. A physiological model of the FB-OOC was characterized by the maintenance of standard cell morphology, cell viability, development of a junctional barrier, and production of baseline cytokine levels. Oxidative stress inducer CSE was used to create a pathologic disease state characterized by increased glutamate dysfunction, increased pro-inflammatory cytokine production, and a shift in glial activation status documenting “neuroinflammation” on-chip.

Next, we evaluated the effect of direct vs indirect exposure to PBDE in the context of the placenta-fetal brain interface. Direct exposure to PBDE at the fetal BBB induced a proinflammatory cytokine response; however, no glial cell activation was detected. Meanwhile, PBDE fileted through the placenta induced endothelial cell toxicity (i.e., less viability and inflammation). However, the fetal brain is protected from neuroinflammation. However, glutamate dysfunction was detected in both indirect and direct treatments of PBDE. This is an interesting phenomenon that needs to be investigated further, as glutamate dysregulation leads to neuronal excitotoxicity and neuroinflammation and can be associated with ASD. These cellular level changes are often associated with the development of many neurological disorders, specifically autism spectrum disorders. Further studies investigating PBDE exposure at the placenta-fetal brain interface and its ability to induce cellular level changes associated with autism spectrum disorders will be evaluated.

Our results highlight that more complex models are necessary to unravel the underlying effect of environmental toxicants. 2D cell culture methodologies do not accurately model the fetal brain, nor the context of the *in-utero* space, to evaluate the effect of maternal exposure to toxicants. Such scientific questions should be evaluated within the perspective of the fetal-maternal interfaces (i.e., placenta and fetal membrane [fetal]-decidua [maternal]) as well as the BBB. The passage of compounds through placental models will allow researchers to investigate the effect of the parent compound, metabolites, generated of cytokines, hormones, growth factors, and neurotransmitters and their combined biological effect on the fetal brain. Incorporating the endothelial-pericyte barrier within neuronal models is important, as the protective effect of the BBB is critical to evaluating a biological question in a physiological context.

The lack of an adequate model and the inability to test within the biological context is a contributing factor to the lack of therapeutics. Currently, magnesium sulfate is the only therapeutic with established neuroprotective effects for preterm births before 32 weeks of gestation that can be administered antenatally (Chollat et al., 2018). Magnesium has anti-inflammatory properties and has been demonstrated to reduce pro-inflammatory cytokines like TNF-alpha (Mazur et al., 2007; Cho et al., 2015). Additionally, magnesium has been shown to decrease extracellular glutamate under ischemic conditions, possibly reducing excitotoxicity (Kang et al., 2011). Other posited mechanisms include limiting calcium influx through voltage-gated channels, reducing apoptosis, and non-competitive NMDA receptor antagonism preventing excitotoxic calcium-induced injury (Nowak et al., 1984; Türkyilmaz et al., 2002). Despite the benefits of Magnesium sulfate being documented since the late 1990s and its clinical use, the definite mechanism remains unclear (Chollat and Marret, 2018). Furthermore, despite its recognized benefits, the only therapeutic indication is in patients at risk of preterm delivery before 32 weeks gestation. No other antepartum therapies exist for neuroprotection despite the plethora of ongoing research. Models such as the one here can help generate data to determine magnesium sulfates’ mechanism of action in the context of the placenta and fetal brain interface.

Approximately 90% of the drug candidates that enter clinical trials fail to gain regulatory approval and reach the market (Sun et al., 2022). Organ-on-chips can provide more physiologically relevant models for preclinical drug testing compared to traditional cell cultures or animal models as they can mimic the complex 3D architecture and functionality of human organs, allowing for more accurate prediction of drug effects in humans. In the context of pregnancy and fetal *effects in utero,* it is important to unravel the maternal, placental, and fetal contribution to the processing of toxicants and therapeutic agents. Specifically, the placenta acts as a selective barrier between maternal and fetal circulations, helping to metabolize and filter substances utilizing drug-metabolizing enzymes, including cytochrome P450 enzymes, glucuronosyltransferases, and glutathione transferases (Mohammed et al., 2020). Utilizing multiple OOC, PLA-OOC, and FB-OOC demonstrated the impact of placental PBDE metabolism byproducts that resulted in a differential response. Future characterization of these metabolites can better illuminate their contribution to fetal neuroinflammation to improve the chances of developing successful therapeutics.

To our knowledge, this is the first study attempting to validate a physiological and pathological model of the fetal BBB in an OOC. One of the primary advantages of our FB model is its ability to recapitulate the complex *in vivo* environment of the developing human brain. By incorporating relevant cell types and extracellular matrix components, our model provides a platform to study the cellular and molecular mechanisms underlying neuroinflammation in a controlled and physiologically relevant manner. While our OOC model represents a significant advancement in the field of neurodevelopmental research, it is important to acknowledge its limitations. Firstly, although astrocytes and microglia in our model are fetal derived, the remainder of the cells are not. Therefore, the response of the endothelium could differ. However, other fetal derived cell lines will be incorporated in the future. Establishment of a barrier can be confirmed in future experiments by using various markers of tight junction (i.e., VE-cadherin and N-cadherin). Dextran propagation, at different time points and varies sizes or transendothelial electrical resistance can be used to determine the functional capacity of the barrier. Once barrier function is further tested the effects of disease states on the barrier function is of vital importance. This model also misses the complete complexity of the fetal brain structures. This can be remedied in future studies by using additional cell lines and oligodendrocytes. Additionally, in future experiments adjusting device design can be altered, shortening the length of microchannels, and may facilitate closer interactions between various cell types. Nonetheless, the validation of a physiological and pathological FB-OOC provides a platform and experimental design to study fetal neuroinflammation. It demonstrates an experiment model utilizing multiple OOCs in series to get closer to a physiologically relevant model.

## Data Availability

The original contributions presented in the study are included in the article/supplementary material, further inquiries can be directed to the corresponding author.
